# Dipicolinic Acid Release by Germinating *Clostridium difficile* Spores Occurs through a Mechanosensing Mechanism

**DOI:** 10.1128/mSphere.00306-16

**Published:** 2016-12-14

**Authors:** Michael B. Francis, Joseph A. Sorg

**Affiliations:** Department of Biology, Texas A&M University, College Station, Texas, USA; Centers for Disease Control and Prevention

**Keywords:** *Clostridium difficile*, DPA, cortex, dipicolinic acid, germination, mechanosensing, osmolytes, *spoVAC*, spore

## Abstract

Classically, dormant endospores are defined by their resistance properties, particularly their resistance to heat. Much of the heat resistance is due to the large amount of dipicolinic acid (DPA) stored within the spore core. During spore germination, DPA is released and allows for rehydration of the otherwise-dehydrated core. In *Bacillus subtilis*, 7 proteins are encoded by the *spoVA* operon and are important for DPA release. These proteins receive a signal from the activated germinant receptor and release DPA. This DPA activates the cortex lytic enzyme CwlJ, and cortex degradation begins. In *Clostridium difficile*, spore germination is initiated in response to certain bile acids and amino acids. These bile acids interact with the CspC germinant receptor, which then transfers the signal to the CspB protease. Activated CspB cleaves the cortex lytic enzyme, pro-SleC, to its active form. Subsequently, DPA is released from the core. *C. difficile* encodes orthologues of *spoVAC*, *spoVAD*, and *spoVAE*. Of these, the *B. subtilis* SpoVAC protein was shown to be capable of mechanosensing. Because cortex degradation precedes DPA release during *C. difficile* spore germination (opposite of what occurs in *B. subtilis*), we hypothesized that cortex degradation would relieve the osmotic constraints placed on the inner spore membrane and permit DPA release. Here, we assayed germination in the presence of osmolytes, and we found that they can delay DPA release from germinating *C. difficile* spores while still permitting cortex degradation. Together, our results suggest that DPA release during *C. difficile* spore germination occurs though a mechanosensing mechanism.

**IMPORTANCE** *Clostridium difficile* is transmitted between hosts in the form of a dormant spore, and germination by *C. difficile* spores is required to initiate infection, because the toxins that are necessary for disease are not deposited on the spore form. Importantly, the *C. difficile* spore germination pathway represents a novel pathway for bacterial spore germination. Prior work has shown that the order of events during *C. difficile* spore germination (cortex degradation and DPA release) is flipped compared to the events during *B. subtilis* spore germination, a model organism. Here, we further characterize the *C. difficile* spore germination pathway and summarize our findings indicating that DPA release by germinating *C. difficile* spores occurs through a mechanosensing mechanism in response to the degradation of the spore cortex.

## Abstract (precis)

*Clostridium difficile* is transmitted between hosts in the form of a dormant spore, and germination by *C. difficile* spores is required to initiate infection, because the toxins that are necessary for disease are not deposited on the spore form. Importantly, the *C. difficile* spore germination pathway represents a novel pathway for bacterial spore germination. Prior work has shown that the order of events during *C. difficile* spore germination (cortex degradation and DPA release) is flipped compared to the events during *B. subtilis* spore germination, a model organism. Here, we further characterize the *C. difficile* spore germination pathway and summarize our findings indicating that DPA release by germinating *C. difficile* spores occurs through a mechanosensing mechanism in response to the degradation of the spore cortex.

## INTRODUCTION

*Clostridium difficile* is a Gram-positive, spore-forming, strict anaerobe that most commonly infects immunocompromised or antibiotic-treated hosts. Many antibiotics have a broad spectrum of activity that disrupts the normal microbiota, which provides colonization resistance to *C. difficile* infection (1). This disruption enables *C. difficile* to colonize and cause disease (2). In a host, *C. difficile* secretes two toxins (TcdA and TcdB) that damage the colonic epithelium and elicit the primary symptoms of disease (3). Though disease is caused by vegetative cells, it is the spore form that makes possible the transition between the aerobic environment and hosts (4).

Spores are metabolically dormant forms of bacteria that are resistant to many harsh conditions (e.g., heat, desiccation, antibiotics) (5, 6). The spore structure is conserved across most spore-forming, Gram-positive bacteria and is important for maintaining the spore’s resistance properties. Contained within the spore core is genomic DNA bound by small acid-soluble proteins (SASPs) and a large quantity of Ca-dipicolinic acid (DPA) (7, 8). The SASPs protect the DNA from UV damage, and DPA packaging helps protect the core from heat by excluding water (7, 9, 10). Surrounding the core is an inner spore membrane, where many of the Ger-type germinant receptors found in *Bacilli*, and most *Clostridia*, are located (6). Surrounding the inner membrane is a thin layer of germ cell wall peptidoglycan and a thick layer of specialized cortex peptidoglycan. In cortex peptidoglycan, many of the *N*-acetylmuramic acid residues have been converted to muramic-δ-lactam residues and are the targets for cortex-degrading enzymes (8). An outer membrane surrounds the cortex and functions as a scaffold with which to build the coat layer. In some spore-forming bacteria, including *C. difficile*, an additional exosporium layer surrounds the spore coat (6).

Spores remain metabolically dormant until specific signals, termed germinants, are detected by receptors (germinant receptors) in the spore. Germinant recognition by germinant receptors leads to the irreversible initiation of the germination process. In *B. subtilis*, a model organism for studying sporulation and germination, the l-alanine germinant is recognized at the inner spore membrane by the GerA germinant receptor (which is composed of GerAA-AB-AC proteins), while l-asparagine, d-glucose, d-fructose, and K^+^ ions (AGFK) are recognized by GerB and GerK (which are composed of GerBA-BB-BC and GerKA-KB-KC, respectively) (8). The activation of these germinant receptors triggers the release of cations and DPA from the core. The mechanism of DPA release is unclear; however, the proteins encoded by the *spoVA* operon (in shorthand, SpoVAA-AB-AC-AD-AEa-AEb-AF) play a role (11–16). In *B. subtilis*, the release of DPA activates the spore cortex lytic enzyme (SCLE) CwlJ, and the actions of CwlJ and SleB lead to cortex degradation (8). This mechanism of spore germination is similar across most spore-forming bacteria studied to date.

*C. difficile* spore germination is triggered by a combination of certain bile acids and amino acids (5, 17–20). In contrast to the mechanisms of germination observed in *Bacilli* and most *Clostridia*, *C. difficile* does not encode the Ger-type germinant receptors (21). Instead, *C. difficile* uses the germination-specific, pseudoprotease CspC as the bile acid germinant receptor (22). In *Clostridium perfringens*, CspA, CspB, and CspC are active proteases with the potential to cleave the SCLE, pro-SleC, to its active form (5, 23–28). Interestingly, *C. difficile* CspA and CspC are pseudoproteases, as their catalytic triads are not complete. Due to the apparent lack of catalytic activity, we proposed a working model where activated CspC signals CspB to cleave pro-SleC to an active form. SleC activation initiates cortex degradation. Recently, another protein, GerS, was identified to play an important role during *C. difficile* spore germination (29). Spores lacking GerS fail to degrade cortex but still process SleC into its active form (29). In contrast to the mechanisms of germination observed in *B. subtilis*, during *C. difficile* spore germination the DPA contained within the core is released after cortex degradation begins (30). Whereas *B. subtilis* releases DPA through a pore presumably formed by the proteins encoded by the *spoVA* operon, *C. difficile* does not encode the entire operon (21, 31). Instead, *C. difficile* encodes three homologues: *spoVAC*, *spoVAD*, and *spoVAE* (31). In *B. subtilis*, the *spoVA* proteins are required for the completion of sporulation, likely due to defects in DPA packaging (32); SpoVAD is thought to act as a DPA binding protein (11), helping to package DPA in the core during spore formation. Moreover, a recent study identified a mobile genetic element that carries *spoVAC*, *spoVAD*, and *spoVAE*, and the resulting overexpression of the proteins encoded by these genes led to accumulation of up to 50% more DPA in the spores and increased heat resistance (10).

*B. subtilis* SpoVAC has been shown to have mechanosensing properties (13). If SpoVAC functions as a mechanosensing protein in *C. difficile*, it could respond to the changes in osmolarity observed at the inner spore membrane due to the removal of constraints placed upon the dormant, dehydrated core by the cortex layer. Cortex degradation may allow pores to open in response to the lower osmotic pressure of the environment relative to that in the DPA-rich core. We hypothesized that a spore germinating in an environment with an osmolyte concentration equal to or higher than that of the core would affect DPA release. Here, we investigated the role high osmolyte concentrations play in cortex degradation and DPA release during *C. difficile* spore germination. We found that high osmolyte concentrations can block DPA release from the core while permitting cortex degradation. Our data suggest that DPA release during germination by *C. difficile* spores is due to changes in osmolarity that occur during cortex degradation.

## RESULTS

### Measuring DPA content of SpoVAC-deficient spores.

**FIG 1:**
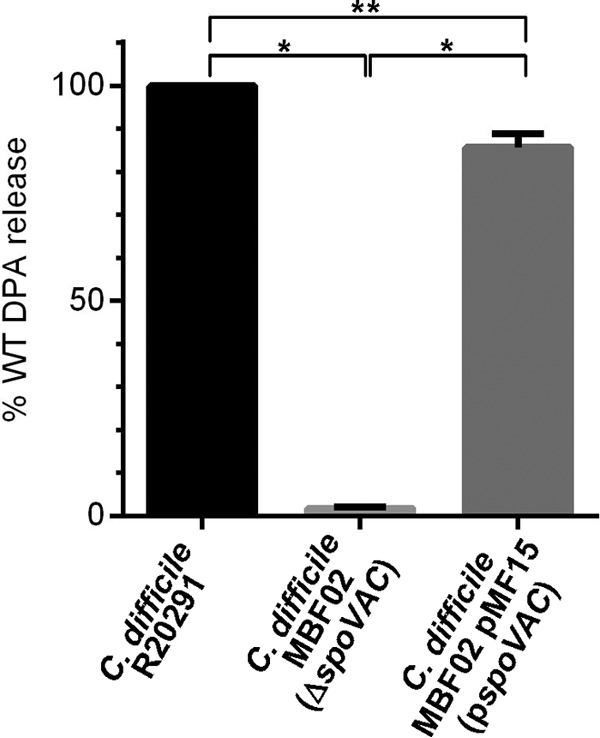
SpoVAC is important for DPA packaging. Equal amounts of spores derived from *C. difficile* strains R20291 (WT), MBF02 (Δ*spoVAC*), and MBF02 pMF15 (p*spoVAC*) were boiled for 15 min, and the amount of DPA was quantified based on Tb^3+^ fluorescence. The data shown represent the average results from three independent experiments, and the error bars represent the standard deviations from the means. All values are reported as the percentage release relative to that of *C. difficile* R20291. *, *P* < 0.001; **, *P* < 0.05.

In a previous study, we determined that cortex degradation precedes DPA release and we hypothesized that SpoVAC may trigger the SpoVA channel to release DPA in a mechanosensing fashion (30). Recently, a mutation in *spoVAC* was shown to affect packaging and release of DPA from the *C. difficile* spore (31). Using the CodA-based allelic exchange system (33), we created a mutant strain in which the *spoVAC* sequence was truncated, with only the first 30 bp and last 30 bp of *spoVAC* present. Spores were prepared from wild-type *C. difficile* R20291, *C. difficile* MBF02 (Δ*spoVAC*), and *C. difficile* MBF02 pMB15 (p*spoVAC*). The DPA content of these spores was measured by boiling the spores, a condition that artificially releases the stored DPA (22). Similar to the results of a recent study (31), spores derived from the *spoVAC* mutant strain contained approximately 1% of the DPA content found in the wild-type strain (Fig. 1), and this could be complemented by expressing *spoVAC* in *trans*. These results confirmed prior observations indicating that *spoVAC* is important for packaging DPA during spore formation (31).

### High sorbitol concentrations delay the onset of *C. difficile* spore germination.

**FIG 2:**
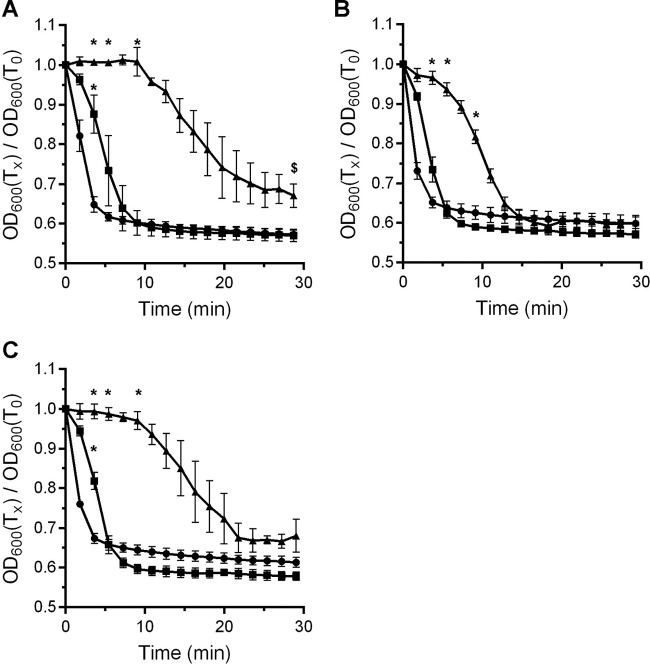
Osmolytes delay germination by *C. difficile* spores. *C. difficile* R20291 spores were germinated in buffer supplemented with taurocholate and glycine in the absence of sorbitol (filled circles) or in buffer supplemented with 19% (filled squares) or 38% (filled triangles) osmolyte (sorbitol [A], trehalose [B], or sucrose [C]). For clarity, every fifth data point is plotted, and the data represent the average results from three independent experiments. Error bars represent the standard deviations from the means. *, *P* < 0.01; $, *P* < 0.05.

To test the effects of osmolytes on *C. difficile* spore germination, we measured the change in the optical density at 600 nm (OD_600_) of spores suspended in either buffer supplemented with taurocholate (TA) and glycine or buffer supplemented with TA, glycine, and an osmolyte (either sorbitol, trehalose, or sucrose). The OD germination assay is a simple method for observing how germination proceeds on the whole (the change from a phase bright spore to a phase dark spore) but does not give detailed information about individual steps (e.g., DPA release, cortex degradation). When *C. difficile* R20291 spores were suspended in buffer supplemented with both TA and glycine, a rapid decrease in the OD_600_ of the suspension was observed. However, when *C. difficile* R20291 spores were suspended in buffer supplemented with TA, glycine, and increasing amounts of sorbitol (0%, 19%, and 38%), the wild-type spores demonstrated a significant delay in the drop in the OD_600_ (Fig. 2A), suggesting that the increasing osmolyte concentration blocked or slowed *C. difficile* spore germination.

To understand if the above observations were due to sorbitol alone or if other osmolytes could substitute for sorbitol, we measured germination in trehalose (Fig. 2B) or sucrose (Fig. 2C). As observed for germination in the presence of sorbitol, *C. difficile* spores germinating in the presence of 19% trehalose or sucrose had a short delay in germination. However, *C. difficile* spore germination was significantly delayed in the presence of 38% trehalose or sucrose (Fig. 2B and C, respectively). These results suggest that *C. difficile* spore germination can be delayed under increased osmolyte concentrations.

### Osmolytes block DPA release during *C. difficile* spore germination.

**FIG 3:**
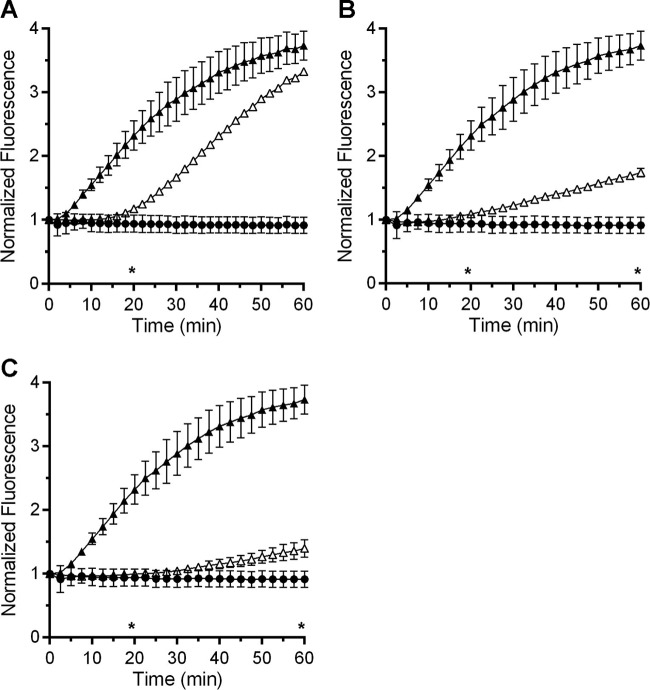
Osmolytes delay DPA release by *C. difficile* spores. *C. difficile* R20291 spores were suspended in buffer containing terbium chloride and glycine alone (closed circles) or supplemented with taurocholate (filled triangles) or with taurocholate and 38% osmolyte (open triangles) (sorbitol [A], trehalose [B], or sucrose [C]). For clarity, every fifth data point is plotted, and the data represent the average results from three independent experiments. Error bars represent the standard deviations from the means. *, *P* ≤ 0.01. Statistical significance for TA plus glycine conditions compared to 38% sorbitol conditions was only tested at the 20- and 60-min time points.

To understand if the osmolyte-mediated delay in germination by *C. difficile* spores was specific to the OD assay conditions or if we would observe similar changes when measuring DPA release, we monitored DPA release by germinating spores in the presence of TbCl_3_ (34, 35). During germination, the released DPA complexes with Tb^3+^, which results in fluorescence of the lanthanide metal. Thus, DPA release by germinating spores is measured in real time by monitoring DPA-dependent Tb^3+^ fluorescence. *C. difficile* R20291 spores were added to buffer supplemented with TA and glycine alone or TA, glycine, and sorbitol, and DPA release was monitored as a readout for germination (Fig. 3A). In the absence of sorbitol, spores rapidly released their stored DPA and an increase in Tb^3+^ fluorescence was observed (Fig. 3A). Interestingly, spores delayed the release of DPA in the presence of sorbitol. We found that 38% sorbitol led to a delay of ~8 min over the course of the experiment (Fig. 3A). To understand if DPA release could be blocked or delayed by other osmolytes, spores were germinated as described above but sorbitol was replaced with trehalose (Fig. 3B) or sucrose (Fig. 3C). Both trehalose and sucrose delayed the DPA release by germinating *C. difficile* spores.

Prior reports have suggested that there is heterogeneity between different *C. difficile* strains in terms of their response to germinants (36–38). Though the rates and efficiencies with which *C. difficile* spores germinate in response to bile acids have been reported, the mechanisms underlying the response to germinants are likely conserved among strains (30, 38, 39). To ensure that our observation that high osmolyte concentrations delay the release of DPA from germinating *C. difficile* spores was not due to a strain-specific phenotype, we determined whether the *C. difficile* M68 strain yielded similar results. DPA release by *C. difficile* M68, ribotype 017, spores was delayed by increased sorbitol or trehalose or sucrose concentrations (see Fig. S1 in the supplemental material). These results suggest that the effects of high osmolyte concentrations on *C. difficile* spore germination are not strain specific and that high osmolyte concentrations delay the release of DPA from the core of germinating *C. difficile* spores.

10.1128/mSphere.00306-16.1 Figure S1  Osmolytes delay DPA release by *C. difficile* M68 spores. *C. difficile* M68 spores were suspended in buffer containing terbium chloride and glycine alone (filled circles) or supplemented with taurocholate (filled triangles) or taurocholate and 38% osmolyte (open triangles) (sorbitol [A], trehalose [B], or sucrose [C]). For clarity, every fifth data point is plotted, and the data represent the average results from three independent experiments. Error bars represent the standard deviations from the means. Download Figure S1, TIF file, 0.8 MB. Copyright © 2016 Francis and Sorg. 2016 Francis and Sorg This content is distributed under the terms of the Creative Commons Attribution 4.0 International license.

### Pro-SleC is cleaved to an active form in high osmolyte concentrations.

**FIG 4:**
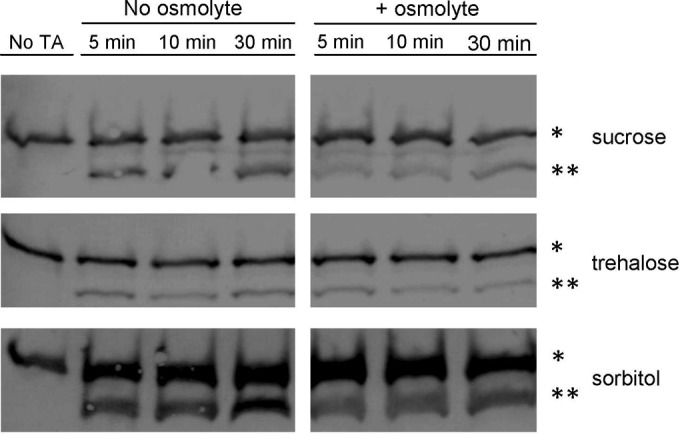
High osmolyte concentrations do not affect SleC activation. Cleavage of pro-SleC (*) to SleC (**) was assayed in buffer supplemented with glycine or buffer supplemented with glycine and taurocholate, with or without 38% osmolyte, at 5, 10, and 30 min.

The delays in OD change and in DPA release could be the result of the high osmolyte concentration retarding the rate at which germinants (TA and glycine) interact with the germinant receptors. To test if the germinants were still activating germination in the presence of osmolyte, we tested if the SCLE pro-SleC was cleaved to its active form (SleC activation is necessary for cortex degradation). *C. difficile* spores were suspended in buffer with glycine only, as a negative control, or in buffer supplemented with TA and glycine with or without 38% osmolyte. Samples were taken at the indicated times and processed for immunoblotting. In the absence of TA, spores did not activate pro-SleC (Fig. 4). However, spores rapidly cleaved pro-SleC to its active form in response to TA and glycine. Significantly, the presence of osmolyte had no effect on the timing of SleC activation (Fig. 4).

### Detecting cortex degradation in the presence of osmolytes.

**FIG 5:**
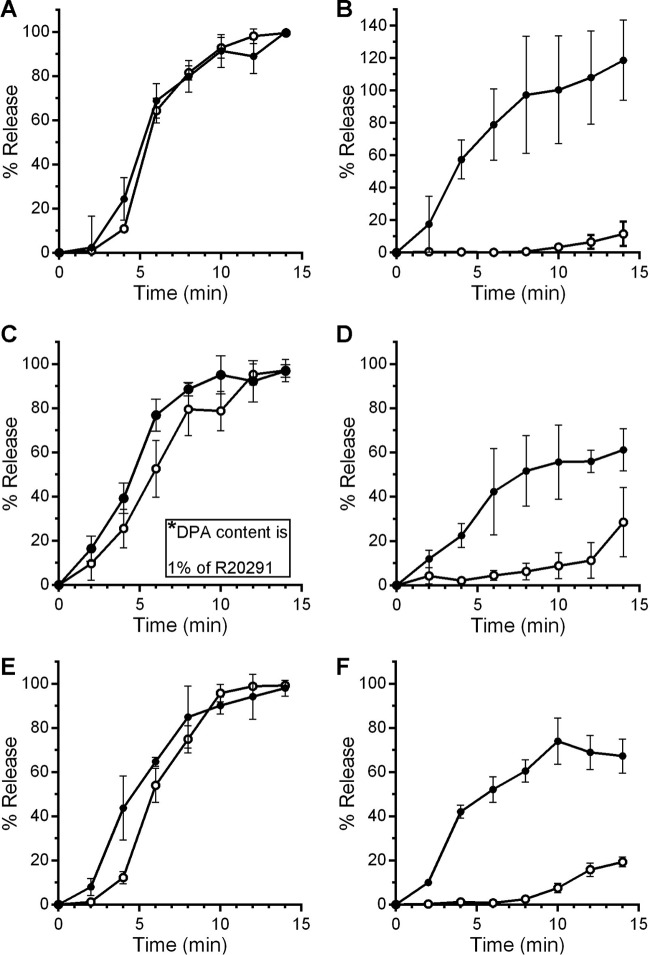
High osmolyte concentrations delay DPA release but not cortex degradation. Purified *C. difficile* R20291 spores (A and B), *C. difficile* MBF02 (Δ*spoVAC*) spores (C and D), or *C. difficile* MBF02 pMF15 (p*spoVAC*) spores (E and F) were suspended in buffer supplemented with taurocholate and glycine and no sorbitol (A, C, and E) or the same buffer supplemented with 38% sorbitol (B, D, and F). At the indicated time points, samples were taken for the amount of cortex fragments (filled circles) and DPA (open circles). Values for all graphs were normalized to the average maximum amount of cortex or DPA released by each strain in the absence of sorbitol. The data represent the average results from three independent experiments, and error bars represent the standard deviations from the means.

If SleC is activated in the presence of osmolyte, the appearance of cortex fragments in the germinant solution should be readily detected. To detect these fragments, we made use of a colorimetric assay that we, and others, have used to quantify the presence of reducing sugars (27, 28, 30, 40). This assay detects the presence *N*-acetylglucosamine (NAG) and *N*-acetylmuramic acid residues (reducing sugars) released during the degradation of cortex peptidoglycan (40). Purified spores were germinated in the presence of TA and glycine and were sampled every 2 min for the presence of both cortex fragments and DPA (Fig. 5). The results for *C. difficile* R20291 spores were similar to those previously reported for *C. difficile* strain UK1, as cortex degradation and DPA release seemingly occurred simultaneously (Fig. 5A) for a wild-type strain (using mutant strains, we previously observed that cortex degradation precedes DPA release [30]). When we tested cortex degradation and DPA release in buffer supplemented with 38% sorbitol with TA and glycine (sorbitol was the only osmolyte tested in this assay because it is the only compound among those we tested that does not generate a reducing end), the release of cortex fragments occurred in a similar manner as in the absence of sorbitol, while DPA release was delayed and only began to be evident after 8 min (Fig. 5B).

When germinated in TA with glycine, *C. difficile* MBF02 (Δ*spoVAC*) spores released cortex fragments similarly to the wild-type strain (Fig. 5C). DPA release by this strain also occurred; however, the total amount of DPA from the MBF02 strain was only 1% of that found with the R20291 strain (Fig. 1). When the *spoVAC* mutant spores were germinated in the presence of 38% sorbitol, the release of what little amount of DPA was present was delayed to the very end of the time period, while cortex degradation remained largely unaffected (Fig. 5D). These observations could be complemented by expressing *spoVAC* in *trans* from a plasmid (Fig. 5E and F).

### Alteration of the osmolyte concentration affects DPA release during germination.

**FIG 6:**
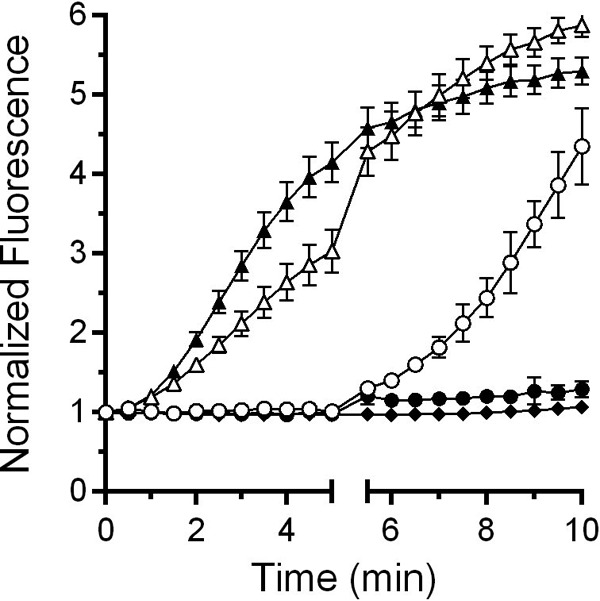
Altering osmotic conditions during germination influences DPA release. Purified *C. difficile* R20291 spores were suspended in buffer supplemented with taurocholate and glycine alone (filled triangles) or also in the presence of 38% sorbitol (filled diamonds) and allowed to germinate for 10 min. In a separate experiment, spores were germinated for 5 min in buffer supplemented with taurocholate and glycine alone (open triangles) or with 38% sorbitol (open circles). These samples then were diluted with 2 volumes of buffer supplemented with taurocholate and glycine alone. Finally, a separate set of spores were germinated for 5 min in buffer supplemented with taurocholate, glycine, and 38% sorbitol (filled circles), and then 2 volumes of 38% sorbitol-containing germination buffer were added and germination was allowed to continue for another 5 min.

If DPA release is dependent on both cortex degradation and a mechanosensing mechanism, changing the osmolyte concentration during germination should result in a marked change in the rate of DPA release. To test the effects of an osmotic downshift on DPA release by germinating spores, spores were allowed to germinate for 5 min in 1 volume of 38% sorbitol-containing germination buffer (containing TA and glycine). Then, either 2 volumes of germination buffer with sorbitol (leading to no change in osmotic conditions) or 2 volumes of the germination buffer without sorbitol (leading to an osmotic downshift) were then added to the well. Germination was then monitored under these new conditions. In samples that had sorbitol-containing germination buffer added, DPA release was delayed, as expected; the osmotic strength of the solution did not change (Fig. 6). However, diluting the osmolyte to 12.7% (a 3-fold dilution) resulted in a rapid release of DPA from the core (Fig. 6). As controls, samples in which the spores were germinated in germination buffer alone or germination buffer supplemented with 38% sorbitol for the duration of the experiment were included. As expected, in the control samples DPA was released in the no-sorbitol germination buffer, while the sorbitol-containing germination buffer blocked DPA release (Fig. 6). These results suggest that DPA release during germination by *C. difficile* spores occurs through a mechanosensing mechanism that is dependent on degradation of the spore cortex layer.

## DISCUSSION

The release of DPA from the core of a spore is a crucial step during spore germination. However, the mechanisms of DPA release by germinating spores are poorly understood. Here, we reaffirmed that SpoVAC plays a critical role in DPA import into the core during spore formation (Fig. 1) (31). Unfortunately, our current understanding of the mechanism of DPA release and SpoVAC activity prevents the separation of DPA packaging during *C. difficile* spore formation from DPA release during germination. Thus, to begin to understand the mechanisms of DPA release by germinating *C. difficile* spores, we raised the concentration of osmolyte in the germination solution. The increase in osmolyte concentration led to a marked delay in DPA release but not a delay in the activation of SleC, a key step in germination (Fig. 3 and 4). By using an assay that detects the presence of reducing sugars (i.e., cortex fragments) (40), we were able to compare when the cortex was degraded relative to DPA release under conditions where the osmotic strength of the medium was increased. Under these conditions, DPA release was delayed though cortex degradation was unaffected (Fig. 5). Because cortex degradation precedes DPA release during *C. difficile* spore germination, these results suggest that cortex degradation relieves the constraints put in place by the cortex on the core, leading to production of the mechanosensing protein, SpoVAC, and permitting DPA release.

*spoVAC* is conserved across most endospore-forming organisms, and the proteins encoded by the *spoVA* operon play important roles during spore formation and germination (10, 31, 32, 41). In *B. subtilis*, formation of a dormant, heat-resistant spore is dependent on 5 of the proteins encoded by the *spoVA* operon: SpoVAA, SpoVAB, SpoVAC, SpoVAD, and SpoVAEb. SpoVAF and SpoVAEa localize to the inner membrane of the spore and have minor roles during spore germination (12). Defects in these proteins reduce germinant-dependent germination but do not affect non-nutrient-mediated spore germination (e.g., exogenous DPA) (12). In *B. subtilis*, the release of DPA from the core triggers degradation of the spore cortex (8). For DPA to be released from the spore core, a signal first must be transmitted from the germinant receptors to the SpoVA protein. SpoVAF and SpoVAEa may play accessory roles during germination to efficiently receive and transmit the germination signal from GerA or GerB/GerK to the SpoVA complex and permit DPA release. Importantly, *C. difficile* does not encode *ger*-type germinant receptors, nor does *C. difficile* encode *spoVAF*, but it does encode *spoVAE* and *spoVAD* homologues (21). The roles of SpoVAE and SpoVAD during *C. difficile* spore germination are unknown. However, based on work with *B. subtilis*, it is likely that SpoVAD is essential for DPA packaging during sporulation (11).

Of the *spoVA*-encoded proteins, SpoVAC is nearly universally conserved among spore formers (41). Though the number of proteins encoded by the *spoVA* operon varies between organisms, nearly all encode *spoVAC*. However, there are a few exceptions. Among the *Clostridiales*, *Bryantella formatexigens* only encodes SpoVAA and SpoVAB, and *Carboxydibrachium pacificum* does not encode any apparent *spoVA* homologues (41). The nearly universal conservation of *spoVAC* among spore formers suggests that mechanosensing plays an important role during spore germination.

Mechanosensing during spore germination was previously studied during germination by *B. subtilis* spores (42–44). Those authors found that a *B. subtilis* Δ*mscL* Δ*mscS* mutant strain germinated similarly to a wild-type strain in response to l-alanine and dodecylamine (43). Thus, the proteins required for osmotic stability during growth play little to no role during germination by *B. subtilis* spores. However, the mechanosensing SpoVAC protein plays an important role during spore formation and germination (31, 32).

The data supporting SpoVAC as a mechanosensing protein are based partly on the ability of recombinant-expressed SpoVAC-myc-His_6_ to protect *Escherichia coli* from an osmotic downshift (13). Those authors found that SpoVAC-myc-His_6_ protected *E. coli* to a similar extent as MscL, a well-studied mechanosensing membrane protein (45, 46). The authors further characterized the protein in conductance studies and found that, when recombinant-expressed in *E. coli* and embedded in lipid vesicles, SpoVAC had a pore size of 4.6 Å (13). This pore size should be large enough to accommodate DPA (a planar molecule with dimensions of 5.2 Å in length and 3.5 Å in width), but the authors mentioned that the vesicles may not recapitulate the lipid content or the hydration state of the inner spore membrane, and this could affect gating of the protein (13). Prior work suggested that the inner spore membrane is in a gel-like state (or in a state that prevents mobility of a protein within the membrane) (47). An interesting hypothesis is that upon the initiation of cortex degradation, local changes at the inner membrane are observed which trigger SpoVAC-mediated DPA release from the core. Subsequently, the change in hydration state of the inner spore membrane at the site of DPA release results in the signal propagating the surrounding SpoVAC proteins. The prediction of such a system would be that high osmolyte concentrations can only block DPA release for so long before a few SpoVAC proteins become activated, randomly. This then would trigger the rest of the SpoVAC proteins to open and would allow DPA to escape the core. In support of this hypothesis, we found that sorbitol, trehalose, or sucrose did not permanently block the release of DPA from germinating spores (Fig. 3; see also Fig. S1 in the supplemental material). These osmolytes only delayed the release of DPA from the germinating spore. In sorbitol-containing germination buffer, the rate with which DPA was released after the initial delay was similar to the rate observed in the absence of sorbitol (Fig. 3A). This suggests that the SpoVAC channel activity is not affected by sorbitol, or water activity, in our assays. In this experiment, sucrose functioned better than trehalose and trehalose functioned better than sorbitol, but DPA release still occurred and the rate of DPA release began to increase at later time points. Importantly though, what little DPA was present in the *C. difficile* Δ*spoVAC* strain was still released during spore germination (Fig. 5C), though the value is presented as 100% release, the data were normalized between conditions with and without sorbitol and not between strains. The Δ*spoVAC* mutant has a low amount of DPA (Fig. 1) (31). This suggests that another protein could function in an accessory role to permit DPA release in response to the change in osmolarity at the inner spore membrane upon cortex degradation, or that the membranes leak DPA in the absence of SpoVAC.

Understanding the process of germination and how DPA release is triggered is likely more straightforward in *C. difficile* than in other spore-forming organisms. Because only *spoVAC*, *spoVAD*, and *spoVAE* are encoded in *C. difficile*, the mechanism of DPA release likely does not involve interaction with other factors (e.g., germinant receptors). In *C. difficile*, the bile acid germinant receptor CspC likely transmits the bile acid signal to CspB, which then cleaves pro-SleC to an active, cortex-degrading form (22, 48, 49). Activated SleC then begins degrading the cortex, allowing expansion and SpoVAC-mediated DPA release, core hydration, and subsequent outgrowth of a vegetative cell. The absence of germinant receptor in the inner spore membrane simplifies the mechanism of DPA release but may result in less specificity and a tendency for an increased amount of spontaneous DPA release. Interestingly, *Clostridium perfringens* encodes a *spoVA* system similar to that of *C. difficile* and also encodes functional germinant receptors (5, 41, 50, 51). How DPA release is signaled in this organism is unknown, but understanding its role would be of important value for understanding how the SpoVAD, SpoVAC, and SpoVAE proteins interact in such a system.

## MATERIALS AND METHODS

### Bacteria and strains.

Wild-type *C. difficile* R20291 and *C. difficile* M68 were routinely grown at 37°C in an anaerobic atmosphere (10% H_2_, 5% CO_2_, 85% N_2_) on brain heart infusion agar supplemented with 5 g/liter yeast extract and 0.1% l-cysteine (BHIS). *E. coli* DH5α, *E. coli* HB101(pRK24), and *B. subtilis* BS49 strains were grown on Luria-Bertani (LB) medium supplemented with antibiotics as needed. Chloramphenicol (20 μg/ml), thiamphenicol (10 μg/ml), kanamycin (50 μg/ml for *C. difficile*, 20 μg/ml for *E. coli*), or tetracycline (5 μg/ml for *C. difficile*, 20 μg/ml for *B. subtilis*) was added where indicated.

### Molecular biology.

Using the *codA*-dependent allelic exchange strategy (33), we engineered a deletion of *spoVAC* in *C. difficile* R20291. To do so, we inserted the Tn*916 oriT* into pMTL-SC7215, using primers pMTL_SC_7215_tn916_L (CTAGAGTCGACGTCACGCGTCCATGGAGATCTCGAGTAACATCTTCTATTTTTCCCAAATCCTTAC) and pMTL_SC_7215_tn916_R (GGCCAGTGCCAAGCTTGCATGTCTGCAGGCCTCGAGCTAAAGGGAATGTAGATAAATTATTAGGTAATCTGC) to make pMF12, as described previously (22). *C. difficile* R20291 DNA was used as a template to amplify 1 kb upstream and downstream of the *spoVAC* deletion using primers spoVAC_ndeI_L (AGCTATGACCGCGGCCGCTGTATCCATATGAGTTCAAAATGGAGATGAAGAGGCAAAAGA), spoVAC_LHF_Rev_II (CTAAAACATCTTAAAAATATAATAAATAATGTCTACATATTTTTTATAATTTTTATCCA), spoVAC_xhoI_L (ATGGATAAAAATTATAAAAAATATGTAGACATTATTTATTATATTTTTAAGATGTTTTAGATGAT), and spoVAC_xhoI_R (TGCCAAGCTTGCATGTCTGCAGGCCTCGAGGTTCTTTAAGGTTAAACATCTCTATACCAC). The resulting 1-kb fragments were stitched together using splicing by overlap extension PCR and subcloned into pMF12 digested with NdeI/XhoI, yielding pMF11. The pMF11 plasmid was introduced into *B. subtilis* BS49 by using standard techniques. Subsequently, the pMF11 plasmid was introduced into *C. difficile* R20291 via conjugal transfer from *B. subtilis* BS49 pMF11, as described previously (22). Tetracycline-sensitive, thiamphenicol-resistant (transposon-negative, plasmid-positive) strains were identified. These isolates were then spread on BHIS supplemented with kanamycin and thiamphenicol to enrich for and identify faster-growing single-crossover integrant clones. The larger colonies were then selected and plated on *C. difficile* minimal medium supplemented with 50 μg/ml 5-fluorocytosine (FC). The colonies that formed after 48 h were tested for thiamphenicol sensitivity by culturing and by PCR with primers 5′-catP3 (ATGGTATTTGAAAAAATTGATAAAAATAG) and 3′-catP2 (TTAACTATTTATCAATTCCTGCAATTCG) to confirm loss of the plasmid. To confirm the deletion in *spoVAC*, the colonies were screened by PCR amplification of the *spoVAC* surrounding region, using spoVAC_ndeI_L and spoVAC_xhoI_R. In order to generate the *spoVAC*-complementing plasmid, *C. difficile spoVAC* was amplified using Phusion polymerase with the 5′-spoVAC_Gibson (CATGATTACGAATTCGAGCTCGGTACCCGGGGATCCTAATACTTATGATATGTAGAATAACAAAATATAATAAATATATTACT) and 3′-spoVAC_Gibson (CCAGTGCCAAGCTTGCATGTCTGCAGGCCTCGAGATCTAGTGGTGGTGGTGGTGGTGTAGAGTATTTGCTATCTGTTGAATCGTAT) oligonucleotides. The resulting fragment was cloned via Gibson assembly (52) between the BamHI and XhoI restriction sites of the *B. subtilis*-*C. difficile* shuttle vector pJS116 to generate pMF15. The nucleotide sequences were confirmed before use.

### Spore formation.

*C. difficile* strains were plated on reduced BHIS plates and allowed to grow for 4 days, as described previously (17, 19, 30). On day 4, growth was harvested from two plates by scraping the plates with a disposable inoculating loop and suspended into 1.5-ml microcentrifuge tubes containing 1 ml sterile water. Tubes were stored at 4°C overnight. The next day, the contents of each tube were resuspended through pipetting and immediately centrifuged for 1 min at 14,000 × *g*. The supernatant was removed and the pellet was resuspended in water and centrifuged for 1 min at 14,000 × *g*; this was repeated for a total of 5 times. After resuspending the pellet again in 1 ml of water, the contents of 2 tubes (2 ml total suspension) were carefully layered over 8 ml of 60% (wt/vol) sucrose. This mixture was centrifuged for 20 min at 4,000 × *g*. Subsequently, the supernatant was removed and the remaining pellet was suspended in 1 ml of sterile water. As described above, spores were washed 5 times in sterile water. After the final wash, the supernatant was removed and the purified spore preparations were combined in 1 ml of sterile water. After purification, the resulting spore suspension was phase bright and >99.9% of vegetative cells had been removed.

### Monitoring initiation of spore germination.

The initiation of spore germination was monitored aerobically at 600 nm (the initiation of *C. difficile* spore germination is unaffected by the presence of oxygen). *C. difficile* spore germination was initiated by suspending spores in 50 mM HEPES (pH 7.5), 100 mM NaCl, 100 mM glycine, 10 mM taurocholate, and 19% or 38% (wt/vol) sucrose, trehalose, or sorbitol. Prior to germination, spores were heat shocked for 30 min at 65°C and then placed on ice. Then, 5 μl of spores was diluted into 995 μl of germination buffer and mixed, and the change in the OD_600_ was measured over time.

### Monitoring DPA release.

DPA release was monitored in real time by using terbium fluorescence, as described previously (30, 38). Briefly, an opaque, 96-well plate was prepared with 125-μl aliquots of the germination solutions (see above) supplemented with 800 μM TbCl_3_. A 5-μl sample of a spore suspension (OD_600_, 60) was added to each well, and the DPA release was monitored using a SpectraMax M3 fluorescence plate reader (Molecular Devices, Sunnyvale, CA) (excitation, 270 nm; emission, 545 nm; cutoff, 420 nm [appropriate wavelengths for the DPA-Tb^3+^ complex]).

### Protein extraction and Western blotting.

Spores were allowed to germinate in germination buffer (described above). NuPAGE soluble proteins (e.g., SleC) were extracted from 2 × 10^9^/ml purified spores as described previously (38). Proteins were separated by SDS-PAGE and then transferred for 1.5 h at 0.75 A to an Immobilon-P polyvinylidene difluoride 0.45-µm membrane (Millipore). Subsequently, the membrane was blocked for 1 h at room temperature in Tris-buffered saline (TBS) supplemented with 1% (vol/vol) Tween 20 (TBST) and 5% dried skimmed milk. The membrane was then incubated at room temperature for 1 h with rabbit anti-SleC antisera. After incubation with the primary antibody, membranes were washed thrice in TBST for 20 min each. The membranes were then labeled with goat anti-rabbit IgG (Life Technologies, Inc.) for 1 h at room temperature. The membranes were again washed as described above and then exposed to 4-chloro-1-naphthol–diaminobenzidine in peroxide substrate buffer. To stop reactions, membranes were rinsed with water. The membranes were then photographed under white light.

### Assay of cortex fragment release by germinating spores.

Cortex fragments were detected according to a method previously reported (30, 40). Briefly, *C. difficile* spores were heat activated, as described above, and stored on ice until use. A preinoculation 1.0-ml sample was drawn to serve as a blank for cortex fragment detection, and a separate 100-μl sample was taken as a blank for measuring DPA release. A target spore density (OD_600_) of ~3.0 yielded the best results for detecting cortex fragments, except for MBF02 spores, which required a spore density of ~3.5. A 1.1-ml 0-time point sample was taken immediately after the addition of spores to the germination buffer and centrifuged for 1 min at 14,000 × *g*. Then, 1.0 ml of this sample was transferred to a fresh tube for cortex fragment analysis and 100 μl was taken to monitor the amount of DPA released. This procedure was repeated at selected time points until the experiment was completed. After all time point samples were collected, the samples were frozen at −80°C and lyophilized.

Lyophilized samples were analyzed for cortex fragments as previously described (40).

### Statistical analysis.

Data points represent the means from three independent experiments, and error bars represent standard errors from the means. Statistical analysis between time points, where indicated, was performed using a two-tailed Student’s *t* test.
