# Supplementary figures and images for: Dipicolinic Acid Release by Germinating Clostridium difficile Spores Occurs through a Mechanosensing Mechanism
Source: mSphere. 2016 Dec 14;1(6):e00306-16. doi: 10.1128/mSphere.00306-16 (PMC5156672; doi:10.1128/mSphere.00306-16)

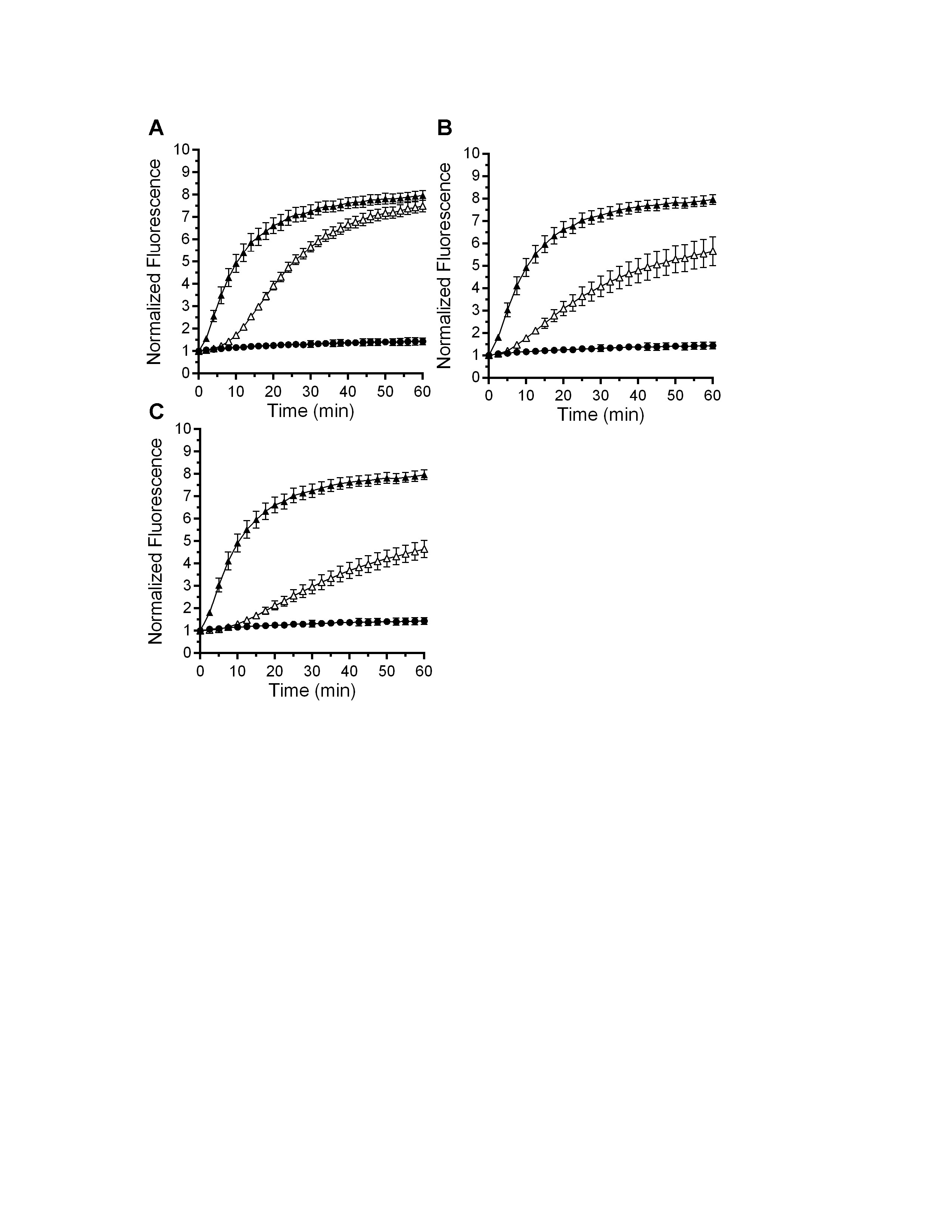

Supplement: Figure S1 [file sph006162207sf1.tif]
